# Hepatitis A among people experiencing homelessness: a case series, Curitiba, Paraná State, Brazil, 2023-2024

**DOI:** 10.1590/S2237-96222026v35e20250865.en

**Published:** 2026-04-17

**Authors:** Janilza Silveira Silva, Renata Barbosa Vilaça Marques de Carvalho, Alcides Souto de Oliveira, Diego Spinoza dos Santos, Liza Regina Bueno Rosso, Leia Regina Silva, Katiuska Ferraz Jansen Negrello, Dirlene Pacheco Venâncio, Monique Boese, Clea Elisa Ribeiro, Cláudia Weingaertner Palm, Tatiane Bartneck Telles, Aroldo José Borges Carneiro, Silvio Luis Rodrigues Almeida

**Affiliations:** 1 Ministério da Saúde, Programa de Treinamento em Epidemiologia Aplicada aos Serviços do SUS, Brasília, DF, Brazil; 2 Secretaria Municipal da Saúde de Curitiba, Centro de Epidemiologia, Paraná, PR, Brazil; 3 Centro de Informações Estratégicas em Vigilância em Saúde de Curitiba, Paraná, PR, Brazil; 4 Prefeitura de Curitiba, Secretaria Municipal da Saúde de Curitiba, Paraná, PR, Brazil

**Keywords:** Hepatitis, Hepatitis A Virus, Homeless Individuals, Disease Outbreaks, Descriptive Epidemiology, Hepatitis, Virus de la Hepatitis A, Personas con Mala Vivienda, Brotes de Enfermedades, Epidemiología Descriptiva

## Abstract

**Objective:**

To describe hepatitis A cases among people experiencing homelessness in Curitiba, Paraná State, Brazil, in 2023-2024.

**Methods:**

This case series included people experiencing homelessness who were reported with hepatitis A in the Notifiable Diseases Information System between November 1, 2023, and May 29, 2024, in Curitiba. This population was identified by reviewing electronic medical records and the registry database of the *Consultório na Rua* [Street Outreach Clinic] team. Sociodemographic, clinical, and laboratory variables were extracted.

**Results:**

Of 281 reported cases, 18 (6.4%) occurred among individuals experiencing homelessness; 16 were male. The mean age was 31±7 years. Regarding substance use in this population, 17 individuals reported crack cocaine use and 14 reported alcohol use. Mean aspartate aminotransferase (AST) and alanine aminotransferase (ALT) levels were 1,290.94 (±1,144.82) U/L and 1,558.13 (±1,050.00) U/L, respectively. The mean total bilirubin level at clinical presentation was 5.29 (±3.59) mg/dL, and jaundice was present in 55.6% of cases. Two deaths were recorded among the 18 cases in individuals experiencing homelessness (mortality: 11.1%), compared with three deaths among the remaining 263 reported cases (mortality: 1.1%).

**Conclusion:**

Symptoms of acute liver failure were observed in hepatitis A cases among people experiencing homelessness, all of whom required hospitalization; deaths were also reported.

## Resumen

**Objetivo:**

Describir los casos de hepatitis A entre personas sin hogar en Curitiba, Paraná, 2023-2024.

**Métodos:**

La serie de casos incluyó a personas sin hogar reportados por hepatitis A en el Sistema de Información de Enfermedades de Notificación Obligatoria entre el 1 de noviembre de 2023 y el 29 de mayo de 2024, en Curitiba, Paraná (Brasil). La identificación de esta población se realizó mediante la revisión de registros médicos electrónicos y la base de datos de registros del equipo de Consultório na Rua. Se obtuvieron variables sociodemográficas, clínicas y de laboratorio.

**Resultados:**

De los 281 casos reportados, 18 (6,4%) ocurrieron en personas sin hogar, 16 de ellas son del sexo masculino. La edad media de los casos fue de 31±7 años. Respecto al uso de sustancias recreativas en esta población, el crack fue reportado por 17 personas; y el alcohol por 14. Las medias de transaminasas oxalacética y pirúvica fueron 1290,94 (± 1144,82) U/L y 1558,13 ( ±1050,00) U/L, respectivamente. La media de bilirrubinemia fue de 5,29 (±3,59) mg/dL en la presentación clínica, y la ictericia estuvo presente en el 55,6% de los casos. Se registraron dos muertes entre los 18 casos en personas sin hogar (letalidad: 11,1%) y tres entre los 263 casos de las otras personas notificadas en el período (letalidad: 1,1%).

**Conclusión:**

Se observaron síntomas de afectación hepática aguda en casos de hepatitis A en personas sin hogar, que tuvieron que ser hospitalizadas; también hubo muertes.

**Ethical aspects**

**This research respected the ethical principles, having obtained the following approval data:**

Research Ethics Committee National Research Ethics Council

Opinion number 7.545.013

Approval date 13/6/2025

Certificate of Submission for Ethical Appraisal: 87956525.0.0000.0008

Registration informed consent Dismissed.

## Introduction

Hepatitis A is an infection caused by the hepatitis A virus (HAV) ^1^. HAV belongs to the genus *Hepatovirus* within the family *Picornaviridae* ^2^ and is the most common cause of acute viral hepatitis worldwide ^3^. Transmission occurs primarily via the fecal-oral route, typically by ingestion of contaminated food or water, but may also result from close person-to-person contact, including specific sexual practices ^1^. The average incubation period of hepatitis A is 28 days, ranging from 15 to 50 days ^4^. 

Clinically, the infection is usually self-limited and asymptomatic in young children. On the other hand, adolescents and adults often experience symptoms such as nausea, vomiting, abdominal pain, fatigue, and fever. As the disease progresses, signs including jaundice, dark urine, and pale stools are common and reflect liver failure, potentially indicating more severe conditions ^4^. Groups at increased risk include travelers, men who have sex with men, individuals who use recreational drugs, and people experiencing homelessness ^3^ ^),(^ ^5^ ^),(^ ^6^.

People experiencing homelessness have been identified as particularly vulnerable to hepatitis A outbreaks, especially in the United States, where recent epidemics have resulted in a high number of hospitalizations and deaths in this population ^7^. In Brazil, although no published reports of hepatitis A outbreaks among people experiencing homelessness were identified, previous studies have documented a high prevalence of other communicable infections-such as human immunodeficiency viruses (HIV), hepatitis B and C, and syphilis ^8^ ^,(^ ^9^ ^)^ -suggesting potential susceptibility to HAV as well.

Comprehensive care for people experiencing homelessness remains a challenge for healthcare systems given their social vulnerability and barriers to accessing services, which may delay timely diagnosis and treatment ^10^ ^),(^ ^11^.

In Curitiba, Paraná State, Brazil, a hepatitis A outbreak occurred between November 2023 and May 2024, predominantly affecting young adults, including people experiencing homelessness, among whom two deaths were confirmed ^12^. Given this context, an investigation was conducted in a partnership between the Field Epidemiology Training Program applied to the Unified Health System Services (EpiSUS-Avançado) and the municipal department of health surveillance. The objective was to describe hepatitis A cases among people experiencing homelessness in Curitiba, diagnosed between November 1, 2023 and May 29, 2024.

## Methods

### Design

This is a case series of hepatitis A conducted in Curitiba, Paraná State, Brazil.

### Background

The city of Curitiba houses 1,773,718 inhabitants ^13^. Between November 1, 2023 and May 29, 2024, the municipality recorded a hepatitis A outbreak, with 281 confirmed cases. Municipal hepatitis A surveillance is conducted via mandatory notification in the Notifiable Diseases Information System (SINAN), with coordination among healthcare services, epidemiological surveillance, and strategies aimed at specific populations, including people experiencing homelessness. During the outbreak, confirmed cases were identified in this population, forming the basis of this investigation. 

### Participants

All confirmed hepatitis A cases reported to SINAN in Curitiba during the study period were included if they were classified as people experiencing homelessness in the healthcare record form. Homelessness status was defined based on information recorded at the time of care that generated the notification, according to the individual’s self-report.

### Variables

The following study variables were defined: sociodemographic (gender, age, race/skin-color, and Health District of residence); clinical presentation (date of hospital admission and signs and symptoms); comorbidities or underlying conditions; laboratory parameters (values recorded on the date of hospital admission); length of hospital stay in days, considering a stay of ≥24 hours in a healthcare facility, in accordance with Resolution No. 2.077/2014 of the Brazilian Federal Council of Medicine ^14^; post-initial care referral and outcome (discharge or death).

### Data sources and measurement

The following data sources were used to identify cases: the hepatitis A database from SINAN; medical records from Emergency Care Units (UPA), accessed via the electronic medical record system of Curitiba (e-Saúde); and the registry database of the *Consultório na Rua* [Street Outreach Clinic] team. A nominal search was conducted for all patients with confirmed hepatitis A in the UPA electronic medical records to identify those who had received care at a UPA up to two months prior disease notification. For these individuals, all visits during this period were reviewed to identify the appointment in which hepatitis A was first suspected or diagnosed. In the corresponding record, homelessness status was assessed by searching for the following keywords: “homeless situation,” “without shelter,” “homeless person,” or “unsheltered.” Additionally, as a complementary strategy, the *Consultório na Rua* team was asked to verify whether individuals diagnosed with hepatitis A during the outbreak had been registered within their program.

Clinical and laboratory variables, as well as recreational substance use, were obtained from requested laboratory test results and medical records. 

### Biases

The following potential biases were considered: selection bias, as the number of cases among people experiencing homelessness may have been underestimated due to the lack of systematic registration of this condition in medical records and information systems; and information bias, since clinical and laboratory data depended on the completeness of available electronic records. To minimize these biases, multiple data sources (SINAN, e-Saúde, and the *Consultório na Rua* registry) were used. Moreover, an active nominal search was conducted across systems, which enhanced case identification and improved data consistency.

### Sample size

The study included all individuals identified via keyword searches in medical records and verification of registration in the *Consultório na Rua* strategy. As this was a descriptive case series including the full universe of cases of interest, no sample size calculation was performed.

### Statistical methods

Absolute and relative frequencies, rates, and measures of central tendency and dispersion were calculated. Data were analyzed and processed using Microsoft Excel 2016 and Epi Info version 7.2.6.0.

## Results

During the study period, 281 hepatitis A cases were confirmed in Curitiba, five of which resulted in death. Two of these deaths occurred among people experiencing homelessness. Based on active searches in electronic medical records and review of the *Consultório na Rua* registry, 18 hepatitis A cases were identified among people experiencing homelessness, comprising the study population.

**Figure 1:**
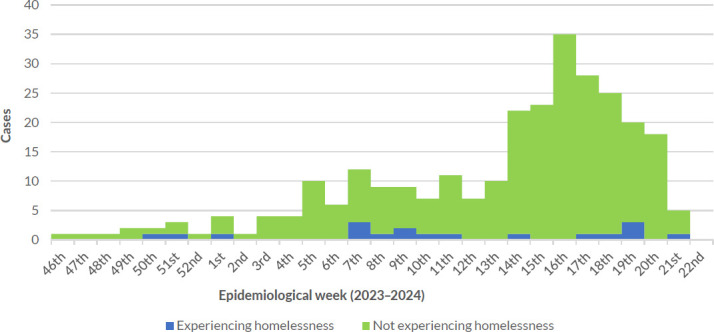
Number of hepatitis A cases among people experiencing homelessness by epidemiological week of symptom onset, Curitiba, Paraná State, Brazil, 2023-2024 (n=281; homelessness n=18)

Of these 18 cases, 16 were male and two were female. The mean age was 31 years (±7). Table 1 shows the sociodemographic characteristics. Hepatitis A cases among people experiencing homelessness were identified from the early weeks of the outbreak (Figure 1).

**Table 1:** Sociodemographic characteristics of people experiencing homelessness diagnosed with hepatitis A. Curitiba, Paraná State, Brazil, 2023-2024 (n=18)

Characteristic	n
**Gender**	
Male	16
Female	2
**Age group**	
20-30	9
31-40	7
41-50	2
**Race/skin-color**	
White	12
Mixed-race	3
Not informed	3
**Health district of residence**	
Boa Vista	2
Boqueirão	2
Cidade Industrial de Curitiba	1
Matriz	8
Portão	3
Santa Felicidade	2

**Figure 2:**
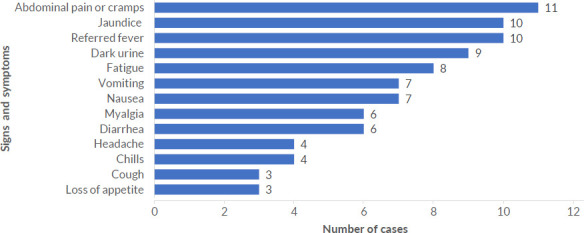
Signs and symptoms of hepatitis A among people experiencing homelessness, Curitiba, Paraná State, Brazil, 2023-2024 (n=18) Note: Multiple possible answers.

All 18 cases remained hospitalized for ≥24 hours at the moment in which hepatitis A infection was suspected or confirmed. The mean length of hospital stay was 4.5 days (±7.2). Among recorded signs and symptoms, abdominal pain was reported in 11 of 18 cases, and jaundice in 10 of 18 cases (Figure 2). A referral following care at an UPA was recorded for 17 of the 18 cases. Of these, three were discharged, five left against medical advice, and nine were transferred to hospitals. For one case, this information was not available in the medical record.

**Figure 3:**
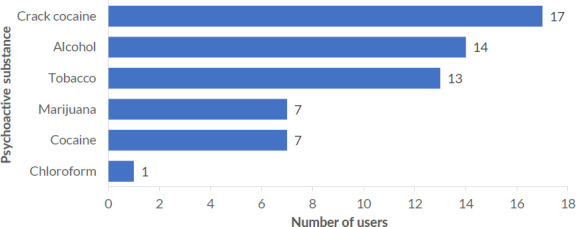
Recreational substance use among people experiencing homelessness with hepatitis A, Curitiba, Paraná State, Brazil, 2023-2024 (n=18) Note: Multiple possible answers.

All individuals reported use of at least one recreational substance, with crack cocaine (17/18) and alcohol (14/18) being the most commonly used (Figure 3).

Laboratory findings showed that people experiencing homelessness presented to the UPA with markedly elevated serum transaminase levels and increased bilirubin concentrations (Table 2).

**Table 2:** Mean and standard deviation (SD) of laboratory parameters of people experiencing homelessness with hepatitis A, Curitiba, Paraná State, Brazil, 2023-2024 (n=18)

Parameter	Mean (±SD)	Laboratory reference
**Hemoglobin (g/dL)**	13.39 (±1.68)	13.5-17.5
**Total leukocytes (mm3)**	8,554.44 (±6,338.38)	3,800-11,000
**Lymphocytes (mm3)**	2,032.83 (±951.66)	760-4,950
**Platelets (mm3)**	231,055.56 (±91,276.87)	150,000-450,000
**Aspartate aminotransferase (U/L)**	1,290.94 (±1,144.82)	5-34
**Alanine aminotransferase (U/L)**	1,558.13^a^ (±1,050.00)	<45^a^
	820.00^b^ (±687.31)	<34^b^
**Gamma Glutamyl Transferase (U/L)**	639.42^a^ (±1,434.75)	12-64^a^
	263.00^b, d^	9-36^b^
**Total bilirubin (mg/dL)**	5.29 (±3.59)	0.2-1.2
**Direct bilirubin (mg/dL)**	4.53 (±2.88)	0-0.5
**Indirect bilirubin (mg/dL)**	1.26 (±0.86)	<1.1
**International Standard Ratio**	1.49 (±0.74)	≤1^c^
**Urea (mg/dL)**	24.50^a^ (±14.21)	18-55^a^
	15.00^b^ (±1.41)	15-43^b^
**Creatinine (mg/dL)**	0.91^a^ (±1.09)	0.72-1.25^a^
	0.51^b^ (±0.08)	0.57-1.11^b^

^a^ Values for males; ^b^ Values for females; ^c^ Laboratory reference for individuals who do not use anticoagulant medications; ^d^ The standard deviation was not calculated because n=1.

During the outbreak, two deaths were recorded among the 18 cases identified among people experiencing homelessness (mortality: 11.1%), compared with three deaths among the 263 cases reported in other individuals during the same period (mortality: 1.1%). 

## Discussion

The investigation of the cluster of hepatitis A cases among people experiencing homelessness in Curitiba revealed a clinical profile characterized by hospitalization (≥24 hours in a healthcare facility) in all cases, clinical manifestations consistent with acute liver failure, and occurrence of fatal outcomes. 

To our knowledge, this is the first study to report a hepatitis A outbreak among people experiencing homelessness in Brazil, using multiple data sources to identify cases. However, the findings should be interpreted with caution in light of certain methodological limitations. These include the potential underestimation of cases due to the lack of systematic documentation of homelessness status in information systems and medical records, as well as incomplete clinical and laboratory data. Additionally, the absence of data on exposure factors precluded a direct assessment of transmission mechanisms within this specific population. 

The markedly elevated mean transaminase levels observed are consistent with acute hepatocellular necrosis, a characteristic finding of hepatitis A virus infection, particularly in adults ^15^. Hyperbilirubinemia, together with the frequent presence of jaundice, reflects impaired biliary excretion and is commonly described in symptomatic presentations of hepatitis A in adults ^16^ ^),(^ ^17^. Alterations in the international normalized ratio indicate impaired liver function with repercussions on the coagulation system. Taken together, the clinical and laboratory findings at initial presentation are consistent with acute hepatitis A with systemic manifestations.

The literature indicates that people experiencing homelessness experience social vulnerabilities that may influence their care trajectories across various health conditions. Factors such as housing instability, food insecurity, and uncontrolled comorbidities have been described as contributing to unfavorable disease progression ^10^ ^),(^ ^11^. These elements help contextualize the setting in which the reported cases occurred, although this study, given its descriptive design, was not intended to evaluate associations between these conditions and the observed outcomes. Most cases presented with jaundice and significant laboratory abnormalities consistent with more advanced disease stages. The documentation of patients leaving care after initial evaluation reinforces the need for strategies that promote engagement and continuity of care.

The frequent alcohol use observed in the reported cases is consistent with studies indicating a high prevalence of alcohol use disorder among people experiencing homelessness in Brazil ^18^. The literature also describes that alcohol-related liver disease may aggravate the clinical course of hepatitis A, an aspect that should be considered when interpreting these findings ^19^.

The results of this study demonstrate the occurrence of cases and deaths among people experiencing homelessness during an urban hepatitis A outbreak. These findings underscore the importance of surveillance strategies that enable timely identification of cases in socially vulnerable populations, as well as coordination between epidemiological surveillance and healthcare services. Hepatitis A vaccination for people experiencing homelessness, particularly in outbreak contexts, should be considered as a preventive measure.

The hepatitis A cases identified among people experiencing homelessness during the outbreak in Curitiba, Paraná State, Brazil, presented with clinical manifestations consistent with acute liver failure, required hospitalization according to the operational criteria adopted, and included recorded deaths during the study period.

## Data Availability

The dataset used in this study is publicly available in anonymized form in the SciELO Data repository, linked to the journal *Epidemiologia e Serviços de Saúde* (RESS), and can be accessed using the identifier: https://doi.org/10.48331/SCIELODATA.HEDWLL. Data should be cited according to the format specified in the repository.
