# Supplementary material for: Hepatitis A among people experiencing homelessness: a case series, Curitiba, Paraná State, Brazil, 2023-2024
Source: Epidemiol Serv Saude. 2026 Apr 17;35:e20250865. doi: 10.1590/S2237-96222026v35e20250865.en (PMC13102379; doi:10.1590/S2237-96222026v35e20250865.en)
Supplement: Translation

# Translation: Hepatite A entre pessoas em situação de rua: série de casos, Curitiba, Paraná, 2023-2024

**DOI:** 10.1590/S2237-96222026v35e20250865.pt

**Authors:** Janilza Silveira Silva, Renata Barbosa Vilaça Marques de Carvalho, Alcides Souto de Oliveira, Diego Spinoza dos Santos, Liza Regina Bueno Rosso, Leia Regina Silva, Katiuska Ferraz Jansen Negrello, Dirlene Pacheco Venâncio, Monique Boese, Clea Elisa Ribeiro, Cláudia Weingaertner Palm, Tatiane Bartneck Telles, Aroldo José Borges Carneiro, Silvio Luis Rodrigues Almeida

**Affiliations:** 1 Ministério da Saúde, Programa de Treinamento em Epidemiologia Aplicada aos Serviços do SUS, Brasília, DF, Brasil; 2 Secretaria Municipal da Saúde de Curitiba, Centro de Epidemiologia, Paraná, PR, Brasil; 3 Centro de Informações Estratégicas em Vigilância em Saúde de Curitiba, Paraná, PR, Brasil; 4 Prefeitura de Curitiba, Secretaria Municipal da Saúde de Curitiba, Paraná, PR, Brasil

**Keywords:** Hepatite, Vírus da Hepatite A, Pessoas Mal Alojadas, Surtos de Doenças, Epidemiologia Descritiva

## Abstract

**Objetivo:**

Descrever os casos de hepatite A entre pessoas em situação de rua em Curitiba, Paraná, 2023-2024.

**Métodos:**

A série de casos incluiu indivíduos em situação de rua notificados por hepatite A no Sistema de Informação de Agravos de Notificação entre 1º de novembro de 2023 e 29 de maio de 2024, em Curitiba, Paraná. A identificação dessa população foi realizada por revisão de prontuários eletrônicos e do banco de cadastros da equipe de Consultório na Rua. Foram extraídas variáveis sociodemográficas, clínicas e laboratoriais.

**Resultados:**

Dos 281 casos notificados, 18 (6,4%) ocorreram em pessoas em situação de rua, sendo 16 delas do sexo masculino. A média de idade dos casos foi de 31±7 anos. Quanto ao uso de substâncias recreativas nessa população, o crack foi referido por 17 pessoas, e o álcool, por 14. As médias de transaminases oxalacética e pirúvica foram de 1290,94 (±1144,82) U/L e 1558,13 (±1050,00) U/L, respectivamente. A bilirrubinemia média foi de 5,29 (±3,59) mg/dL na apresentação clínica, e a icterícia esteve presente em 55,6% dos casos. Foram registrados dois óbitos entre os 18 casos em pessoas em situação de rua (letalidade: 11,1%) e três entre os 263 casos dos demais indivíduos notificados no período (letalidade: 1,1%).

**Conclusão:**

Sintomas de acometimento hepático agudo foram observados nos casos de hepatite A em pessoas em situação de rua, que tiveram que ser internadas; também houve a ocorrência de óbitos.

**Aspectos éticos**

**Esta pesquisa respeitou os princípios éticos, obtendo os seguintes dados de aprovação:**

Comitê de ética em pesquisa Comissão Nacional de Ética em Pesquisa

Número do parecer 7.545.013

Data de aprovação 13/6/2025

Certificado de apresentação de apreciação ética 87956525.0.0000.0008

## Introdução

A hepatite A é uma infecção causada pelo Vírus da Hepatite A (HAV) ^1^. O HAV é membro do gênero *Hepatovirus* e da família *Picornaviridae* ^2^, e é a forma mais comum de hepatite viral aguda ^3^. A transmissão ocorre principalmente pela via fecal-oral, associada ao consumo de alimentos ou água contaminados, mas também pode resultar do contato interpessoal próximo, por meio de práticas sexuais específicas ^1^. O período de incubação médio da hepatite A é de 28 dias, variando de 15 a 50 dias ^4^. 

Do ponto de vista clínico, a infecção costuma ser autolimitada e assintomática em crianças pequenas, mas em adolescentes e adultos pode manifestar-se com sintomas como náuseas, vômitos, dor abdominal, fadiga e febre. Na evolução, sinais como icterícia, colúria e acolia fecal são frequentes e refletem comprometimento hepático, podendo indicar formas mais graves da doença ^4^. Grupos considerados de maior risco incluem viajantes, homens que fazem sexo com homens, indivíduos que usam substâncias recreativas e pessoas em situação de rua ^3^ ^),(^ ^5^ ^),(^ ^6^.

Populações em situação de rua foram identificadas como particularmente vulneráveis a surtos de hepatite A, sobretudo nos Estados Unidos, onde a ocorrência de epidemias recentes resultou em elevado número de hospitalizações e óbitos nessa população ^7^. No Brasil, embora não tenham sido encontrados registros publicados de surtos de hepatite A em pessoas em situação de rua, estudos prévios documentaram alta prevalência de outras infecções transmissíveis, como HIV, hepatites B e C e sífilis ^8^ ^),(^ ^9^, o que sugere uma possível susceptibilidade também ao HAV.

O cuidado integral voltado para as pessoas em situação de rua constitui um desafio para as redes de atenção à saúde, dada a vulnerabilidade social e as barreiras de acesso aos serviços, que podem retardar o diagnóstico e o tratamento oportuno ^10^ ^),(^ ^11^.

Em Curitiba, Paraná, ocorreu um surto de hepatite A, entre novembro de 2023 e maio de 2024, com predomínio de casos em adultos jovens, incluindo pessoas em situação de rua, entre as quais se confirmaram dois óbitos ^12^. Diante desse contexto, realizou-se uma investigação em parceria entre o Programa de Treinamento em Epidemiologia Aplicada aos Serviços do Sistema Único de Saúde (EpiSUS-Avançado) e a vigilância municipal, com o objetivo de descrever os casos de hepatite A em pessoas em situação de rua, em Curitiba, Paraná, diagnosticadas entre 1º de novembro de 2023 e 29 de maio de 2024.

## Métodos

### Delineamento

Trata-se de uma série de casos de hepatite A realizada em Curitiba, Paraná.

### Contexto

Curitiba, Paraná, possui uma população residente de 1.773.718 habitantes ^13^. No período de 1º de novembro de 2023 a 29 de maio de 2024, o município registrou um surto de hepatite A, com a notificação de 281 casos confirmados. A vigilância municipal da hepatite A é realizada por meio da notificação compulsória no Sistema de Informação de Agravos de Notificação (Sinan), com articulação entre os serviços assistenciais, a vigilância epidemiológica e as estratégias voltadas a populações específicas, incluindo pessoas em situação de rua. No contexto do surto de hepatite A no município, foram identificados casos confirmados nessa população, que constituíram o objeto desta investigação. 

### Participantes

Foram incluídos todos os casos confirmados de hepatite A notificados no Sinan no município de Curitiba, Paraná, no período do estudo, classificados como pessoas em situação de rua no registro do atendimento. A condição de pessoa em situação de rua foi definida a partir da informação registrada no atendimento que originou a notificação, conforme autodeclaração do indivíduo.

### Variáveis

Foram definidas as seguintes variáveis de estudo: sociodemográficas (sexo, idade, raça/cor da pele, Distrito Sanitário de procedência); apresentação clínica (data de admissão hospitalar, sinais e sintomas); comorbidades ou condições de base; parâmetros laboratoriais (referentes à data de internação); tempo de internação em dias, considerando como internação a permanência ≥24h no serviço de saúde, conforme resolução n^o^ 2.077/2014 do Conselho Federal de Medicina ^14^; encaminhamento pós atendimento inicial e desfecho (alta ou óbito). 

### Fontes de dados/mensuração

Para a identificação dos casos, foram usadas as seguintes fontes de informação: Banco de dados de hepatite A do Sinan e prontuário de atendimento nas Unidades de Pronto Atendimento, por meio do prontuário eletrônico de Curitiba, Paraná (e-Saúde), além de banco de cadastros da equipe de Consultório na Rua. Foi feita uma busca nominal de todos os pacientes com diagnóstico confirmado de hepatite A no prontuário eletrônico das Unidades de Pronto Atendimento (UPA), com o objetivo de identificar os que tinham sido atendidos em UPA até dois meses antes da notificação da doença. Na sequência, procurou-se, entre os atendimentos realizados nesse período, o atendimento no qual ocorreu a suspeita ou o diagnóstico de hepatite A. No registro desse atendimento, buscou-se identificar se a pessoa estava em situação de rua pesquisando os seguintes termos-chave no prontuário: “situação de rua”, “sem abrigo”, “morador de rua” ou “sem teto”. Além disso, como estratégia complementar, foi solicitado à equipe de Consultório na Rua verificar a existência de cadastro de pessoas com o diagnóstico de hepatite A no contexto do surto na referida estratégia.

As variáveis clínico-laboratoriais e uso de substâncias recreativas foram coletadas dos exames laboratoriais solicitados e dos prontuários de atendimento médico. 

### Vieses

Foram considerados como potenciais vieses: o viés de seleção, dado que o número de casos entre pessoas em situação de rua pode estar subestimado pela ausência de registro sistemático dessa condição em prontuários e sistemas de informação; e o viés de informação, uma vez que os dados clínicos e laboratoriais dependeram da completude dos registros eletrônicos disponíveis. Para minimizar esses vieses, foram utilizadas múltiplas fontes de dados (Sinan, e-Saúde e cadastro do Consultório na Rua), assim como foi realizada uma busca ativa nominal nos sistemas, o que ampliou a identificação dos casos e permitiu maior consistência das informações.

### Tamanho do estudo

O estudo incluiu todos os indivíduos identificados por meio de busca de termos-chave no prontuário do atendimento médico e da verificação de cadastro na estratégia Consultório na Rua. Por tratar-se de um estudo descritivo de série de casos que inclui o universo completo dos casos de interesse, não houve cálculo amostral.

### Métodos estatísticos

Foram calculadas frequências absoluta e relativa, taxas, medidas de tendência central e dispersão. Para a análise e o processamento de dados, usou-se o Microsoft Excel 2016 e o Epi Info versão 7.2.6.0.

## Resultados

No período analisado, foram confirmados 281 casos de hepatite A em Curitiba, Paraná, dos quais, cinco evoluíram para óbito. Dois desses óbitos ocorreram em pessoas em situação de rua. A partir da busca ativa em prontuários eletrônicos e do cadastro da equipe de Consultório na Rua, foram identificados 18 casos de hepatite A em pessoas em situação de rua, que constituíram a população deste estudo.

Dos 18 casos identificados em pessoas em situação de rua, 16 eram do sexo masculino, e dois, do sexo feminino. A média de idade foi de 31 anos (±7). As características sociodemográficas estão descritas na Tabela 1. Ocorreram casos de hepatite A em pessoas em situação de rua desde as primeiras semanas do surto (Figura 1).

**Tabela 1:** Características sociodemográficas das pessoas em situação de rua diagnosticadas com hepatite A. Curitiba, Paraná, 2023-2024 (n=18)

Característica	n
**Sexo**	
Masculino	16
Feminino	2
**Faixa etária**	
20-30	9
31-40	7
41-50	2
**Raça/cor da pele**	
Branca	12
Parda	3
Sem informação	3
**Distrito sanitário de procedência**	
Boa Vista	2
Boqueirão	2
Cidade Industrial de Curitiba	1
Matriz	8
Portão	3
Santa Felicidade	2

**Figura 1:** Número de casos de hepatite A em pessoas em situação de rua, por semana epidemiológica de início de sintomas, Curitiba, Paraná, 2023-2024 (n=281; situação de rua n=18)

Todos os 18 casos permaneceram internados por período igual ou superior a 24 horas no atendimento em que se suspeitou ou confirmou a infecção por hepatite A. A média de permanência hospitalar foi de 4,5 dias (±7,2). Entre os sinais e sintomas registrados, dor abdominal esteve presente em 11 dos 18 casos, e icterícia em 10 das 18 ocorrências (Figura 2). Houve registro de encaminhamento após atendimento em Unidade de Pronto Atendimento (UPA) para 17 dos 18 casos. Desses, três receberam alta, cinco evadiram do serviço e nove foram transferidos para unidades hospitalares. Para um caso, essa informação não estava disponível no prontuário.

**Figura 2:** Sinais e sintomas em pessoas em situação de rua com hepatite A, Curitiba, Paraná, 2023-2024 (n=18) Nota: múltiplas respostas possíveis.

Todas as pessoas faziam uso de pelo menos uma substância recreativa, sendo o crack (17/18) e o álcool (14/18) as mais utilizadas (Figura 3).

**Figura 3:** Uso de substâncias recreativas em pessoas em situação de rua com hepatite A, Curitiba, Paraná, 2023-2024 (n=18) Nota: múltiplas respostas possíveis.

Os parâmetros laboratoriais revelam que as pessoas em situação de rua se apresentaram ao atendimento na UPA com alteração considerável das transaminases séricas e elevação das bilirrubinas (Tabela 2).

**Tabela 2:** Média e desvio-padrão (DP) de parâmetros laboratoriais em pessoas em situação de rua com hepatite A, Curitiba, Paraná, 2023-2024 (n=18)

Parâmetro	Média (±DP)	Referência laboratorial
**Hemoglobina (g/dL)**	13,39 (±1,68)	13,5-17,5
**Leucócitos totais (mm3)**	8.554,44 (±6.338,38)	3.800-11.000
**Linfócitos (mm3)**	2.032,83 (±951,66)	760-4.950
**Plaquetas (mm3)**	231.055,56 (±91.276,87)	150.000-450.000
**Transaminase oxalacética (U/L)**	1.290,94 (±1.144,82)	5-34
**Transaminase pirúvica (U/L)**	1.558,13^a^ (±1.050,00)	<45^a^
	820,00^b^ (±687,31)	<34^b^
**Gama Glutamil Transferase (U/L)**	639,42^a^ (±1.434,75)	12-64^a^
	263,00^b, d^	9-36^b^
**Bilirrubina total (mg/dL)**	5,29 (±3,59)	0,2-1,2
**Bilirrubina direta (mg/dL)**	4,53 (±2,88)	0-0,5
**Bilirrubina indireta (mg/dL)**	1,26 (±0,86)	<1,1
**Razão Normalizada Internacional**	1,49 (±0,74)	≤1^c^
**Ureia (mg/dL)**	24,50^a^ (±14,21)	18-55^a^
	15,00^b^ (±1,41)	15-43^b^
**Creatinina (mg/dL)**	0,91^a^ (±1,09)	0,72-1,25^a^
	0,51^b^ (±0,08)	0,57^a^-1,11^b^

^a^ valores para pessoas do sexo masculino; ^b^ valores para pessoas do sexo feminino; ^c^ referência laboratorial para pessoas que não fazem uso de medicações anticoagulantes; ^d^ não foi calculado o desvio padrão porque n=1.

No contexto do surto, registraram-se dois óbitos entre os 18 casos de hepatite A identificados em pessoas em situação de rua (letalidade: 11,1%) e três óbitos entre os 263 casos registrados nos demais indivíduos notificados no mesmo período (letalidade: 1,1%). 

## Discussão

A investigação do aglomerado de casos de hepatite A em pessoas em situação de rua em Curitiba, Paraná, revelou um perfil clínico caracterizado por internação (≥24h no serviço de saúde) em todos os casos identificados, manifestações clínicas compatíveis com acometimento hepático agudo e ocorrência de desfechos fatais. 

Este estudo é pioneiro ao relatar um surto de hepatite A em pessoas em situação de rua no Brasil, utilizando diferentes fontes de dados para identificação dos casos. No entanto, os resultados devem ser interpretados com cautela, considerando algumas limitações metodológicas. Entre as limitações do estudo, destacam-se a possibilidade de subestimação do número de casos devido à ausência de registros sistemáticos da situação de rua nos sistemas de informação e prontuários médicos, bem como lacunas na completude de dados clínico-laboratoriais. Além disso, a ausência de dados sobre fatores de exposição não permitiu avaliar diretamente mecanismos de transmissão nessa população específica. 

As elevadas médias de transaminases observadas são compatíveis com necrose hepatocelular aguda, achado característico da infecção pelo vírus da hepatite A, particularmente em adultos ^15^. A hiperbilirrubinemia associada à presença frequente de icterícia reflete comprometimento da excreção biliar, frequentemente descrito em apresentações sintomáticas da hepatite A em adultos ^16^ ^),(^ ^17^. As alterações da razão normalizada internacional indicam comprometimento da função hepática com repercussão no sistema de coagulação. Em conjunto, os achados clínicos e laboratoriais observados no primeiro atendimento descrevem um padrão compatível com hepatite A aguda com manifestações sistêmicas.

A literatura aponta que populações em situação de rua vivenciam vulnerabilidades sociais que podem influenciar seu percurso de cuidado em diferentes agravos. Aspectos como instabilidade habitacional, insegurança alimentar e comorbidades não controladas são apontados na literatura como fatores que podem contribuir para evolução desfavorável da doença ^10^ ^),(^ ^11^. Esses elementos ajudam a compreender o cenário em que os casos aqui apresentados ocorreram, embora este trabalho, por seu delineamento descritivo, não tenha por objetivo avaliar relações entre essas condições e os desfechos registrados. A maioria dos casos apresentou icterícia e alterações laboratoriais relevantes condizentes com estágios avançados da doença. O registro de evasões após o primeiro atendimento reforça a necessidade de estratégias que favoreçam o vínculo e a continuidade do cuidado.

O uso frequente de álcool observado nos casos descritos encontra respaldo em estudos que apontam alta prevalência de alcoolismo entre pessoas em situação de rua no Brasil ^18^. A literatura descreve que a hepatopatia alcoólica pode agravar a evolução clínica da hepatite A, devendo esse aspecto ser considerado na interpretação dos achados ^19^.

Os resultados deste estudo evidenciam a ocorrência de casos e óbitos entre pessoas em situação de rua no contexto de um surto urbano de hepatite A. Esses achados reforçam a importância de estratégias de vigilância que permitam a identificação oportuna de casos em populações socialmente vulneráveis, bem como a articulação entre vigilância epidemiológica e serviços assistenciais. A vacinação contra hepatite A em populações de rua, especialmente em contextos de surto, deve ser considerada como medida preventiva.

Em síntese, os casos de hepatite A identificados em pessoas em situação de rua durante o surto em Curitiba, Paraná, apresentaram manifestações clínicas compatíveis com acometimento hepático agudo, internações segundo critério operacional adotado e registro de óbitos no período analisado.

## Data Availability

O conjunto de dados utilizado neste estudo encontra-se disponível, de forma anonimizada, em acesso público no repositório SciELO Data, vinculado à revista Epidemiologia e Serviços de Saúde (RESS), podendo ser acessado por meio do identificador: https://doi.org/10.48331/SCIELODATA.HEDWLL. A referência aos dados deve seguir a citação indicada no próprio repositório.
